# Multi-state MRAM cells for hardware neuromorphic computing

**DOI:** 10.1038/s41598-022-11199-4

**Published:** 2022-05-03

**Authors:** Piotr Rzeszut, Jakub Chȩciński, Ireneusz Brzozowski, Sławomir Ziȩtek, Witold Skowroński, Tomasz Stobiecki

**Affiliations:** 1 Institute of Electronics, AGH University of Science and Technology, Al. Mickiewicza 30, 30-059 Kraków, Poland; 2 Faculty of Physics and Applied Computer Science, AGH University of Science and Technology, Al. Mickiewicza 30, 30-059 Kraków, Poland

**Keywords:** Engineering, Materials science, Mathematics and computing, Nanoscience and technology

## Abstract

Magnetic tunnel junctions (MTJ) have been successfully applied in various sensing application and digital information storage technologies. Currently, a number of new potential applications of MTJs are being actively studied, including high-frequency electronics, energy harvesting or random number generators. Recently, MTJs have been also proposed in designs of new platforms for unconventional or bio-inspired computing. In the current work, we present a complete hardware implementation design of a neural computing device that incorporates serially connected MTJs forming a multi-state memory cell can be used in a hardware implementation of a neural computing device. The main purpose of the multi-cell is the formation of quantized weights in the network, which can be programmed using the proposed electronic circuit. Multi-cells are connected to a CMOS-based summing amplifier and a sigmoid function generator, forming an artificial neuron. The operation of the designed network is tested using a recognition of hand-written digits in 20 $\times$ 20 pixels matrix and shows detection ratio comparable to the software algorithm, using weights stored in a multi-cell consisting of four MTJs or more. Moreover, the presented solution has better energy efficiency in terms of energy consumed per single image processing, as compared to a similar design.

## Introduction

Unconventional computing architectures such as artificial neural networks (ANN) have superior properties over conventional CMOS-based circuits in solving a number of computational problems, e.g., image or voice recognition, navigation, optimization and prediction^1–5^. As a concept, neural networks have been proved to be fast, flexible and energy-efficient. However, their digital implementation uses large amount of resources^6^, which leads to high area needed to implement them. An alternative solution, opposite to the digital implementation, is to use analog-based circuits, where signals are represented as continuous voltage values rather than quantized bits^7–10^. In such implementations, a key component is a programmable resistive element, such as memristor^11^, which can act as a weight in an artificial neuron. While using a solely digital implementation of a neural network may lead to high resource and energy consumption, using mixed digital and analog electronic circuits may enable more compact and energy-efficient solutions. In a number of the proposed analog ANN implementations, neuron behavior was mimicked by a resistive RAM (RRAM) element^12^, which changed its resistance due to the conductor/insulator transition^7^. However, cells based on resistive or phase-change technology suffer from limited durability and may degrade over time and subsequent programming cycles^13^. On the contrary, spintronic elements such as memristors, nano-oscillators^14^ or probabilistic bits^15^, based on magnetic tunnel junctions (MTJs), which rely on magnetization switching or dynamics, do not have such endurance issues, are compatible with the CMOS technology and have been already shown to exhibit superior biomimetic properties^16^. In addition, recent theoretical works have predicted that neural networks are able to work efficiently not only with weights represented by real numbers, but also with binary or quantized values^17–19^.

Recently, we have proposed a design of multi-state spin transfer torque magnetic random access memory (STT-MRAM) cells^20,21^, which may be used in neuromorphic computing schemes as synapses^22–27^ or as a standard multi-state memory unit. In this paper, we present a fully functional hardware implementation design of a neural network, which needs no additional components for operation, except for input and output devices. The design of a single synapse is based on multi-bit STT-MRAM cells forming quantized weights, interconnected with a minimal set of transistors forming amplifiers in the conventional CMOS technology. The entire network is made of neurons arranged in four layers. The operation principle of the proposed neural network is validated using handwritten digits recognition task utilizing MNIST^28^ database. We show that the multi-cell consisting of four MTJs is sufficient for the network to achieve a recognition error rate below 3%, while providing better energy efficiency per operation than circuit presented by Zhang et. al.^29^.

## Experimental

### Mulltibit-cell based artificial synapse

**Figure 1:**
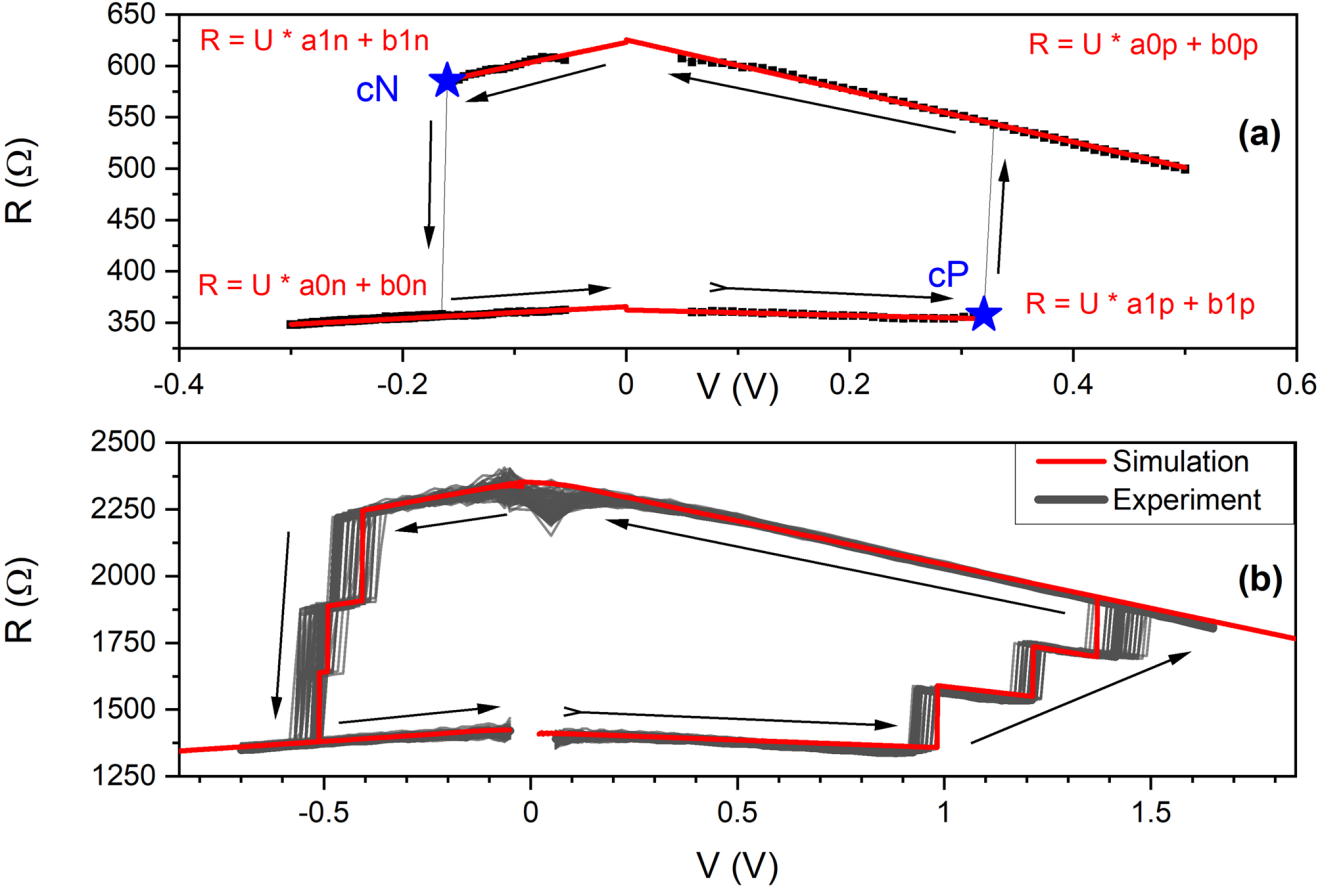
(**a**) Experimental *R*(*V*) dependence (solid points) and the model consisting of four lines and two critical points (stars) presenting a single MTJ behaviour. (**b**) A representative simulation result of three serially-connected MTJs (solid line) together with a series of example measurements (gray-scale lines) of a two-bit multicell. Parameters of a single MTJ were used to model the multi-cell characteristics. Arrows indicate voltage sweep direction.

A key element of the design of the ANN is a spintronic memristor, which involves serially connected MTJs. Each of the MTJs may be characterized by a *R*(*V*) curve (Fig. 1a), where two stable resistance states can be observed, as well as critical voltages (*cN* and *cP*), for which the switching occurs. By serially connecting *N* of such MTJs^20^, a multi-state resistive element is obtained (Fig. 1b), for which $N+1$ resistance states are observed.

**Figure 2:**
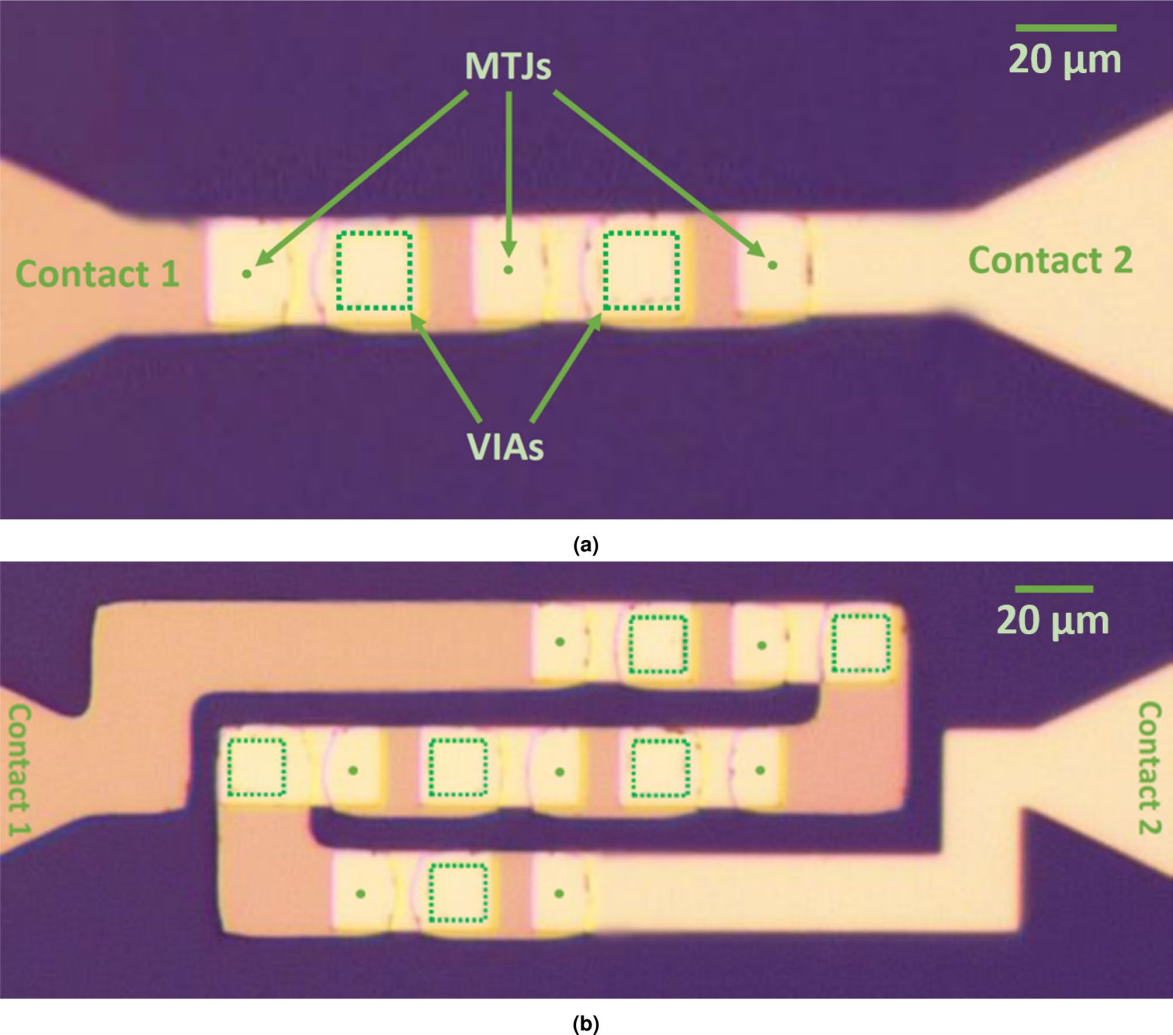
Microimage of the experimental setup: (**a**) three and (**b**) seven serially connected MTJs (dots) with vias (squares) marked.

The concept of the multi-cell was experimentally confirmed using up to seven MTJs connected in series. For the simulation of the network, we introduce a model of the multi-cell based on the following protocol. A typical *R*(*V*) loop of an MTJ may be approximated using four linear functions (resistance vs. bias voltage dependence in each MTJ state) and two threshold points (switching voltages) as presented in Fig. 1a. In addition, in the case of a real MTJ the following parameters are related to each other: $a1n = -a1p = a1$, $b1n = b1p = b1$, $a0n = -a0p = a0$ and $b0n = b0p = b0$. Moreover, a current resistance state (high or low resistance) has to be included. Using such a model of the *R*(*V*) curve allows also to calculate other transport curves, including *V*(*I*). The proposed model corresponds to all MTJs that were investigated during the study. Parameters obtained from the experimental part, and further used in the simulation, are presented in Tab. 1. MTJs with perpendicular magnetic anisotropy were patterned as pillars 100 nm in diameter and interconnected using metalization layers and vias (Fig. 2).

**Table 1:** Expected values and standard deviation of parameters obtained from experiment.

Param.	Unit	$\mu$	$\sigma$
a1	$\mathrm{A}^{-1}$	−310	3
b1	$\Omega$	665	12
a0	$\mathrm{A}^{-1}$	−30	3
b0	$\Omega$	360	12
cN	A	−3.1e−4	1.5e−5
cP	A	8.0e−4	1.5e−5
TMR	%	84	–

**Figure 3:**
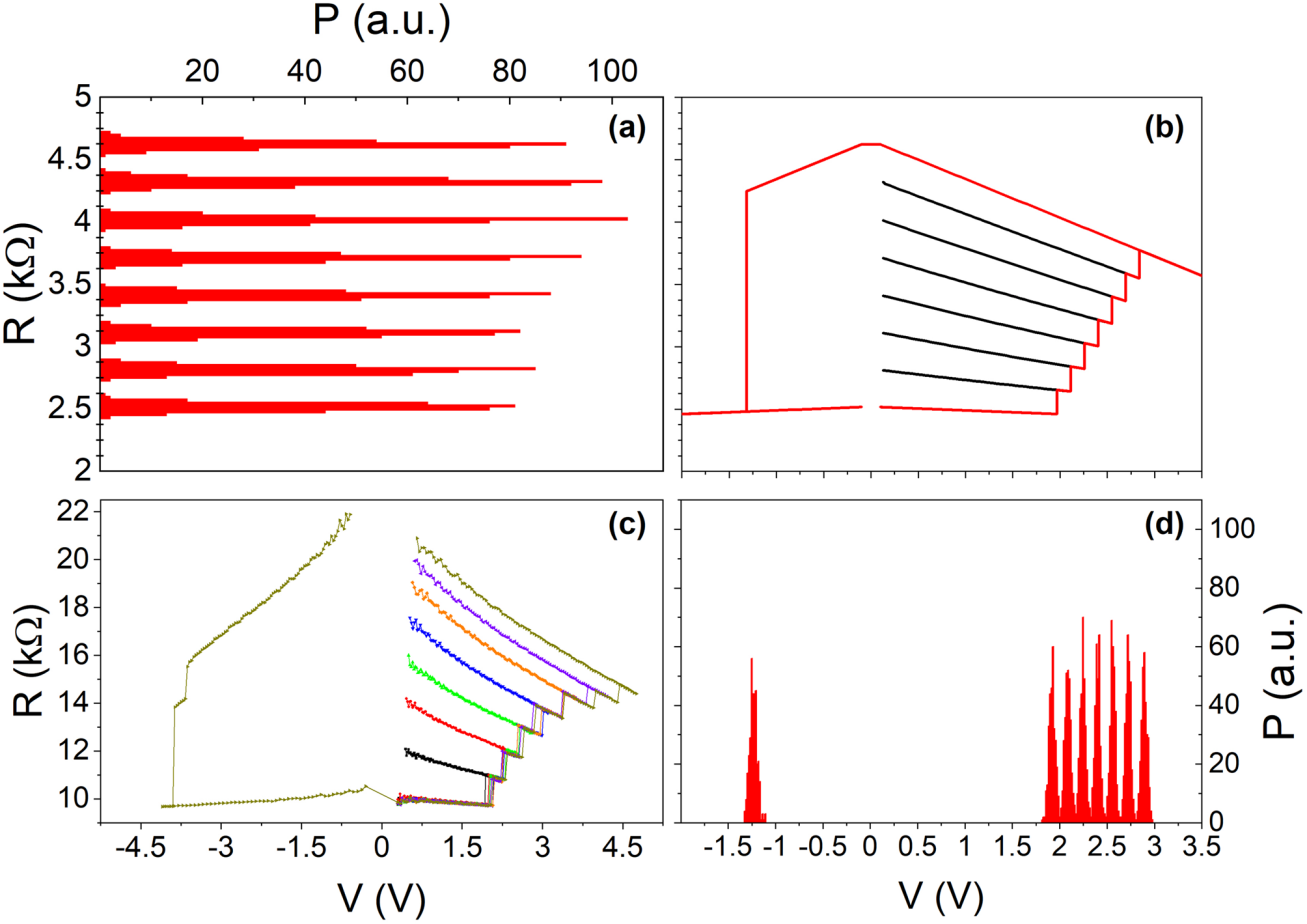
Simulation and experimental results for seven serially connected MTJs with a given parameter spread. (**a**) Spread of readout resistances for the simulation. (**b**) Representative write-read-erase curves. Red line represents full write-read-erase cycle, while black ones represent write-read cycles while programming subsequent values. (**c**) Experimental data for seven serially connected MTJs. MTJs in this case come from another batch, characterized by higher RA product of around ${20}\text{\O}mega \,\,{\upmu \mathrm{m}^{2}}$, which results in approximately three times higher resistance of the multicell and slightly higher switching voltages. (**d**) Spread of all write voltages for the simulation.

The model was used to simulate serially connected MTJs and a representative comparison between simulation and experiment is presented in Fig. 1b. Moreover, simulations of up to seven MTJs were carried out, where, additionally, a spread of parameters was taken into account. This allowed for defining distribution of stable resistance states as well as voltages used for writing. The results of such simulation as well as representative experimental data are presented in Fig. 3.

### Electronic neuron

**Figure 4:**
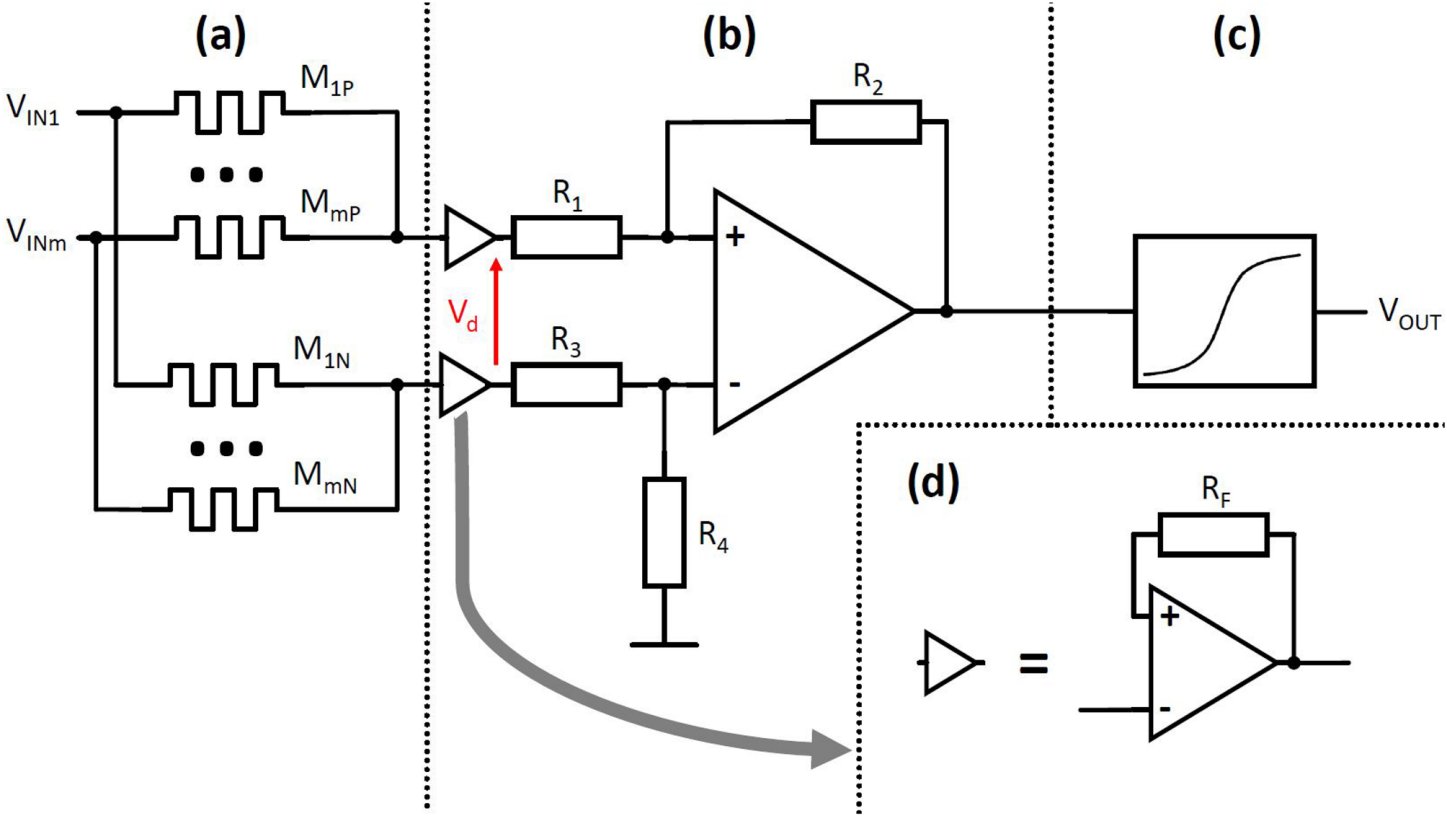
The proposed neuron design with multi-cells. The circuit consists of (**a**) a set of memristors serving as a quantized weight, (**b**) a differential amplifier with voltage followers (**d**) at input and (**c**) a sigmoid function block.

After the analysis of the multi-cell, which may be used as a programmable resistor for performing weighted sum operation for many input voltages, we turn to the artificial neuron design. A schematic diagram of the proposed neuron is presented in Fig. 4. The circuit is powered by a bipolar power supply, where inputs and output ($V_{INm}$, $V_{OUT}$) are provided as bipolar analog signals. To enable positive and negative weights, each of the signal inputs uses a pair of programmable MTJ multi-cells ($M_{mP}$ and $M_{mN}$). In the case when the multi-cell resistances meet the condition $M_{mP} < M_{mN}$, a positive weight is achieved, whereas for the case of $M_{mP} > M_{mN}$ a negative weight value is obtained. An alternative design with multiple MTJs connected in series with a separate select transistors has been proposed recently in Ref.^30^. For equal multi-cell resistances, a zero weight is obtained, which is equivalent to the situation when an input is disconnected from the synapse. The resistive summing circuit architecture is being used in order to implement an addition operation while reducing the footprint of the synapse. A differential amplifier converts differential voltage to a single bipolar signal, which is transformed using a non-linear sigmoid function. This voltage may be used as the input of the next synapse, or as the output of the network. Additionally, to provide a constant bias, a standard input with constant voltage may be used, where the level of this constant bias is determined in the same way as weights for other functional inputs.

### Neural network circuit

The electrical circuit implementing the proposed neural network was designed in a standard CMOS technology—UMC 180 nm. To program the demanded resistance of seven serially connected MTJs, a voltage of about 3.25 V is needed, so input/output (I/O) 3.3 V transistors were used to design a circuit for MTJs programming purpose, while for other circuits, a standard 1.8 V transistors were used. An individual neuron circuit is composed of three parts. At the input, two resistive networks consisting of memristors implement a multiplication of input voltages by coefficients and summing of these products (Fig. 4a). Next, the obtained voltages are subtracted and amplified to the demanded value in a differential amplifier (Fig. 4b). Voltage followers are used to separate stages of the circuit and eliminate unwanted loading (Fig. 4d). Finally, the third part is a sigmoid function block, which implements the activation function (Fig. 4c). It is based on an inverter and has negative transfer characteristic, thus appropriate polarizations of signals are required.

The differential voltage $V_d$ generated by the divider network (Fig. 4a) connected to a pair of voltage followers (Fig. 4d) can be expressed as:

$$\begin{aligned} V_d = -\frac{1}{G_{sum}}\left( \sum _{i=1}^{m}V_{INi}(G_{iP}-G_{iN})\right) , \end{aligned}$$

where:

$$\begin{aligned} G_{iX}= & {} \frac{1}{M_{iX}}\\ G_{sum}= & {} m * G_{ave} = m * \frac{G_{min}+G_{max}}{2} \end{aligned}$$

It can be assumed that sums of all memristors’ conductances in both positive and negative branch are nearly equal, and can be well approximated by the average of minimum ($G_{min}$) and maximum ($G_{max}$) conductances of memristors used, multiplied by the number of inputs (*m*) in the neuron.

## Results

**Figure 5:**
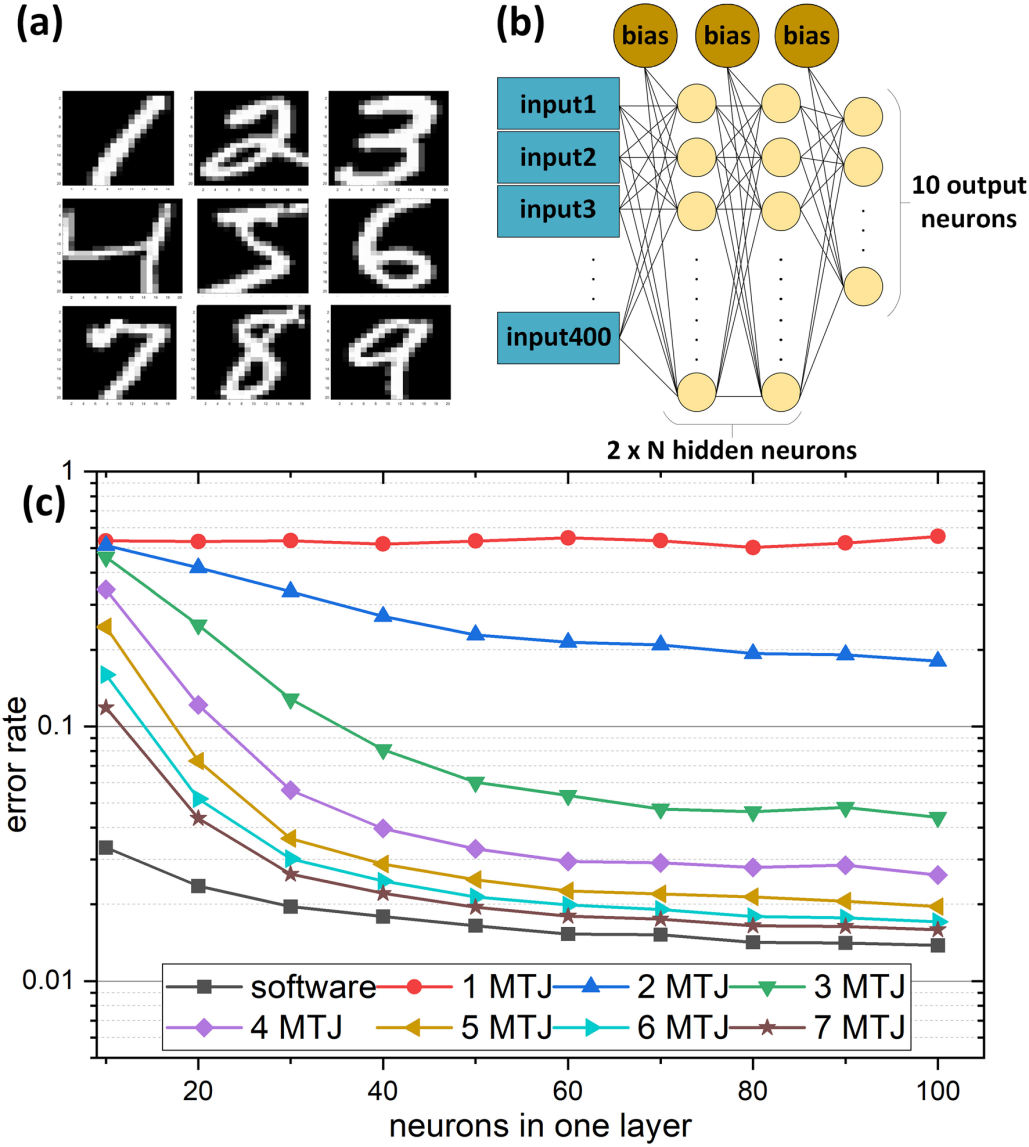
Simulated neural network based on multi-bit MRAM cells. Handwritten digits from MNIST database (**a**) are recognized by a standard neural network with architecture shown in (**b**), where black lines represent network weights and yellow circles represent individual neurons. After training, weights calculated by software are replaced by discretized values corresponding to 1–7 serial MTJs MRAM cells, which affects the network performance (**c**).

To evaluate the performance of the multi-bit MTJ cell-based ANN, a set of classification tasks using the MNIST dataset of handwritten digits (Fig.5a) was prepared. The conceptual architecture used for the network is shown in Fig. 5b and consists of the input layer, two hidden layers containing *N* neurons each and the output layer. A benchmark software network was trained using the standard scaled conjugate gradient method and cross-entropy error metrics, with *tanh* activation function for every layer except the last one, where the *softmax* function was used. Then, its performance was evaluated on a testing subset that has been drawn randomly from the input data and has not participated in training. This procedure was repeated 50 times in total, with training and testing subsets being redrawn each time, leading to an average error estimate for each network size.

Having established the performance of the benchmark software network, the evaluation of our MTJ-based design was performed. The original float-accuracy weights between different neurons were replaced by their discrete versions corresponding to our multi-state MTJ synapses. The new weights were calculated using simulated conductance data (as described in Sec. “Multibit-cell based artificial synapse”) and rescaled by tuning amplifier gains to match the desired value range for the neurons. Then, the performance of the network was re-evaluated on the testing data subset. The results are presented in Fig. 5c. It can be seen that, as long as the number of MTJs used per multi-state cell exceeds three, the performance of the MTJ-based solution is comparable to the original software version, with differences being only incremental in character. Due to a relatively shallow structure of our network, the total number of individual MTJ elements necessary to perform the calculation is thus remarkably low and ranges from around 200 to around 700, depending on the assumed tolerance for error. This is one order of magnitude lower than the number previously reported for quantized neural networks based on MTJs with comparable performance^19^.

The neural network shown in Fig. 5b, using 7 MTJs per memristor, was also described and simulated electrically in Hspice for the same data as computer simulations mentioned above, assuming 7 MTJs per memristor. Input voltages (with maximum amplitude of 0.2 V) corresponding to hand written MNIST digits were changed to a next image every ${4}\,\,{\upmu \mathrm{s}}$. The circuit gave the same results as theoretical calculations—for a given subset of cases the same error rate was achieved. The circuit had a latency of approximately ${1}\,\,{\upmu \mathrm{s}}$ and to process one picture, only 37.4 pJ of energy were needed. It is therefore a significant improvement compared to the work by Zhang et. al.^29^, where processing of a 10 by 10 pixel area (4 times smaller area than our 20 by 20 pixel images) consumed 194 pJ. The power consumption of our network could be further decreased, and speed could be increased at the expense of the output voltage. Also, the total resistance of each synapse might be increased by connecting additional resistances as well as by careful optimization in the MTJ structure such as using devices with higher RA product^31^ and by further miniaturization of the MTJ pillar size below 100 nm in diameter^32^. However, it could also lead to deterioration of the reliability of the ANN.

## Discussion

The presented architecture of full hardware artificial neural network proves to be an effective way of performing neuromorphic computing. Compared to other solutions, it utilizes standard MTJs that are compatible with STT-MRAM technology, which has been recently developed for mass production. Additionally, MTJs in such application are very stable over time and they exhibit high endurance in terms of reprogramming, comparing to low-energy barrier MTJs used in probabilistic computing. To validate the circuit, the artificial CMOS-based neuron was designed, consisting of multi-cell based synapses, differential amplifiers and sigmoid function generator. It was shown that the quantized-weight approach enables the development of a functional artificial neural network, capable of solving recognition problems with accuracy level similar to the benchmark software model. Moreover, the electronic simulations additionally proved low latency of the operation of the order of $\upmu \mathrm{s}$ as well as low energy consumption per recognized picture.

## Methods

### Circuit details

**Figure 6:**
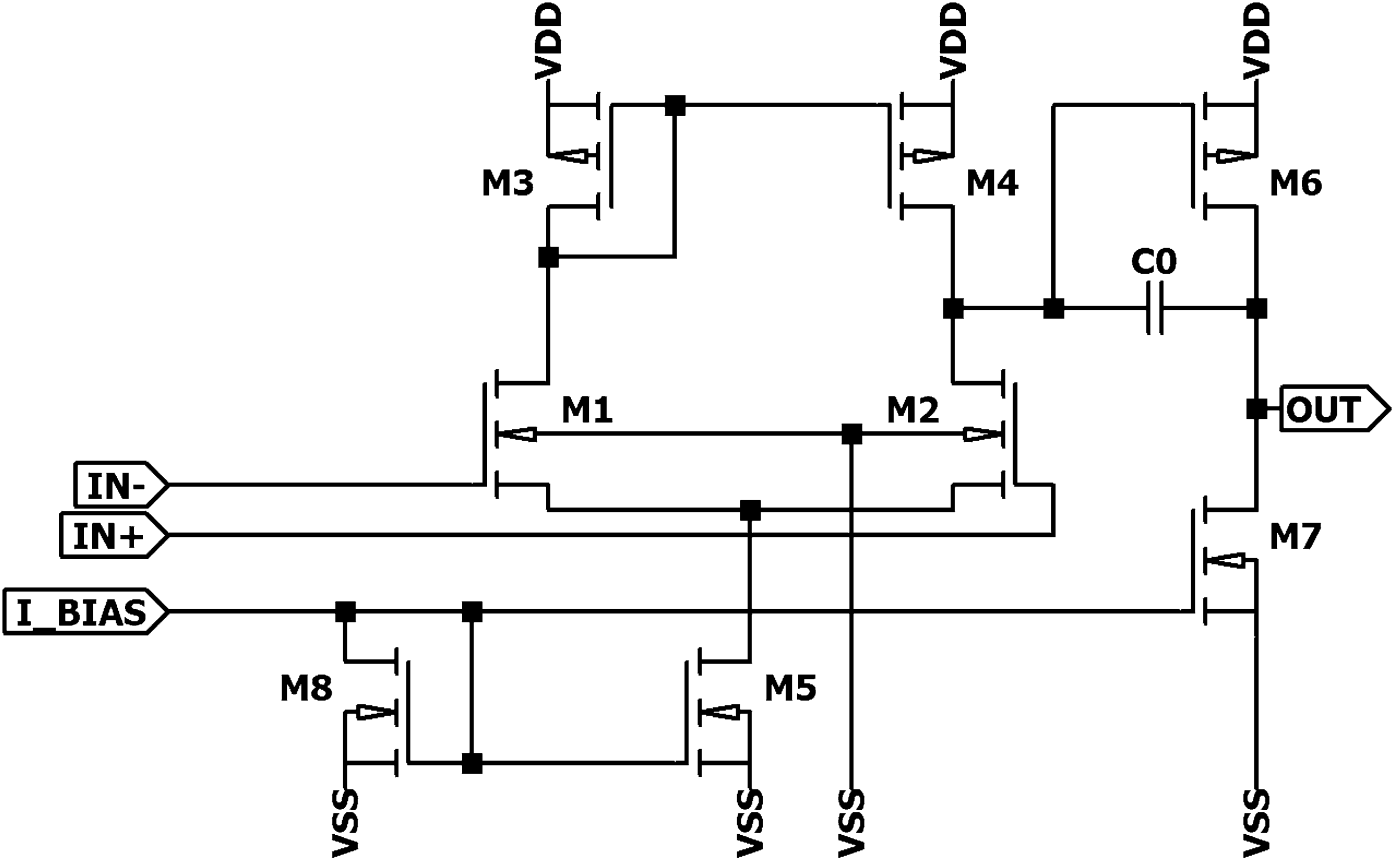
Operational amplifier circuit used in the design.

The operational amplifier, presented in Fig. 6 was designed as a two stage circuit consisting of a differential pair M1, M2 with a current mirror load M3, M4 biased by M5 with a current of ${1}\,\,{\upmu \mathrm{A}}$. The output stage M6, M7 provided appropriate amplification and output current. The total current consumed by the operational amplifier is about ${12}\,\,{\upmu \mathrm{A}}$ and amplification with an open loop of around 74 dB. Dimensions of transistors were chosen in such a way to obtain the smallest area possible while meeting the required electrical parameters (width of M1 and M2 is ${0.7}\,\,{\upmu \mathrm{m}}$, M3 and M4 is ${0.45}\,\,{\upmu \mathrm{m}}$, M5 and M8 is ${0.96}\,\,{\upmu \mathrm{m}}$, M6 is ${7.48}\,\,{\upmu \mathrm{m}}$, and M7 is ${7}\,\,{\upmu \mathrm{m}}$, capacitance of C0 is 100fF).

**Figure 7:**
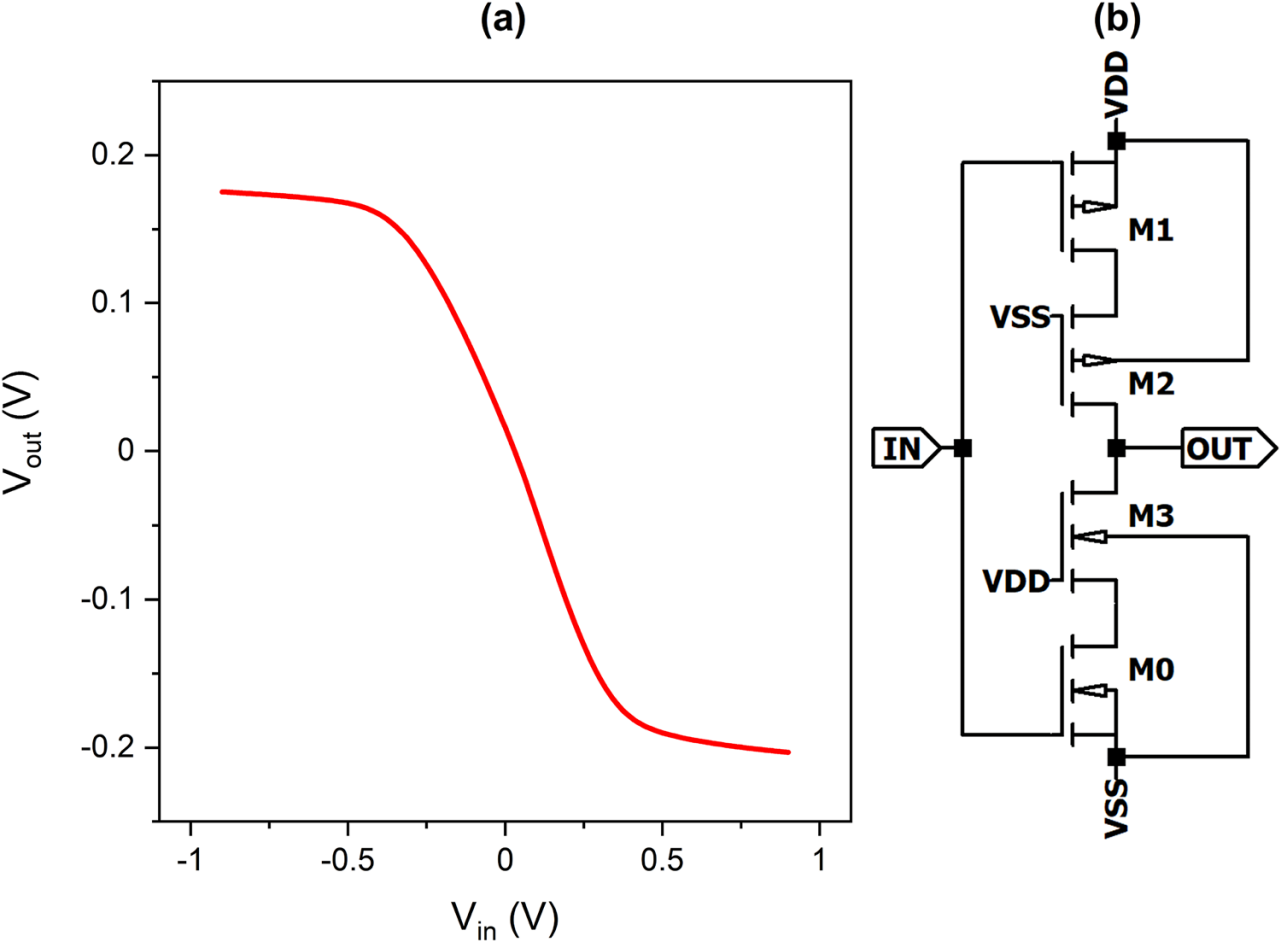
(**a**) A transfer function of a sigmoid-generating inverter implemented by (**b**) the proposed inverter circuit.

The final stage of the neuron is a circuit, which performs activation functions and has negative hyperbolic tangent transfer characteristic, presented in Fig. 7b. It is designed as a modified inverter, which has voltage-to-voltage transfer in contrast to other solutions, such as resistive-type sigmoid^33^. Transistors M2 and M3 work as resistors, moving operating point of transistors M0 and M1 to the linear region. Finally, the circuit implements the transfer characteristic shown in Fig. 7a. Minimum length of channels were used (180  nm, except for M3, which uses 750nm), while their width was chosen to obtain required characteristics and output current necessary to drive the next stage. Therefore, the width of M0 and M3 is ${60}\,\,{\upmu \mathrm{m}}$, M1 is ${228}\,\,{\upmu \mathrm{m}}$, and M2 is ${56}\,\,{\upmu \mathrm{m}}$.

Using Cadence Virtuoso, layouts for amplifier and sigmoid were designed. Dimensions of each circuits are ${17.1 \times 17.4}\,\,{\upmu \mathrm{m}}$ for op-amp and ${17.5 \times 32}\,\,{\upmu \mathrm{m}}$ for sigmoid. Netlists with parasitic elements were extracted for further simulations performed in Hspice.

### Programming of the synapse

**Figure 8:**
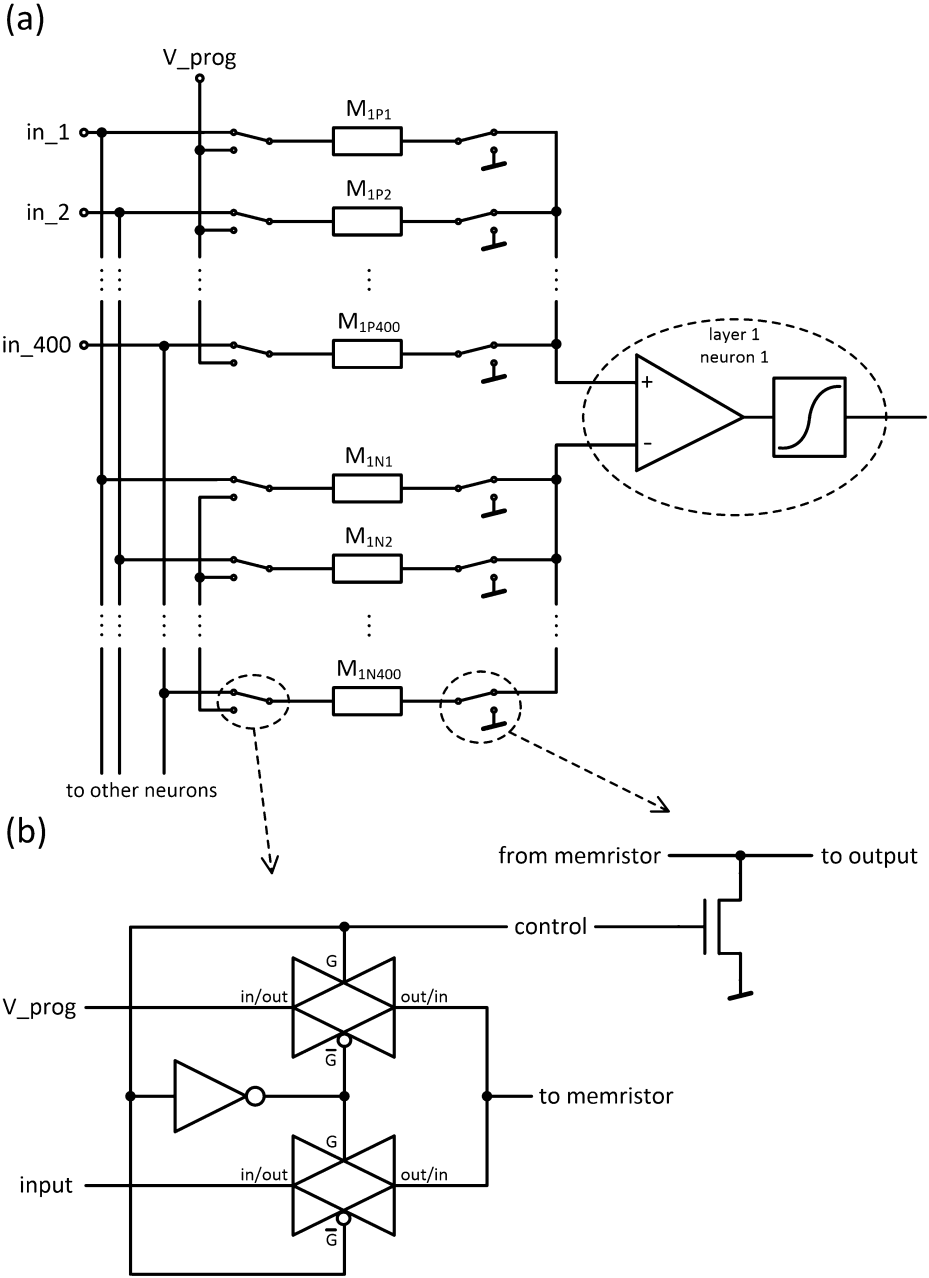
(**a**) Schematic of connection of programming switches and (**b**) the design of the programming switches.

The important part of the design involved a circuit for memristors programming. The overview of the programming circuit is presented in Fig. 8. The switches are controlled from the digital circuit in such a way that the memristor to be programmed is connected with one terminal to the programming voltage input and the other terminal to the ground. After the selected elements are connected, the required voltage value is applied to the programming input in order to program the chosen memristors. Those elements that are not programmed with a given voltage are disconnected from the programming input. In the next cycle, another set of memristors is connected for programming and another voltage is applied. In such solution, all memristors may be programmed in a number of cycles corresponding to the number of stable quantized states of used memristors (e.g., for 7 MTJs per memristor the programming may be completed in only 8 cycles; if the programming voltage spread is too high, additional cycles might be introduced or adaptive programing scheme can be used, however state-of-the-art MTJ industrial fabrication technology can meet requirements with accepted write voltage distribution^34^). The purpose of the digital control circuits to connect the desired components to the programming voltage and ground lines or to switch to normal operation. The state of the switches is stored in serially connected flip-flops. Therefore, additional AND gates controlled by the “enable” signal are used to disconnect all memristors while entering information about elements for programming. Then, after setting the appropriate programming voltage, the enable signal goes high for the duration of programming. The flip-flop and the AND gate are placed as close to the switches as possible, to save connection length. Digital components placed close to the sensitive analog circuit do not have influence on them, because during the operation of the ANN the digital circuitry is inactive, remaining in a static state (no clock signal) while providing the connection of memristors to the analog circuit.
